# Spatial representation of temporal complementarity between three variable energy sources using correlation coefficients and compromise programming

**DOI:** 10.1016/j.mex.2020.100871

**Published:** 2020-03-18

**Authors:** Fausto A. Canales, Jakub Jurasz, Alexander Kies, Alexandre Beluco, Marco Arrieta-Castro, Andrés Peralta-Cayón

**Affiliations:** a Department of Civil and Environmental, Universidad de la Costa, Barranquilla, Atlántico, Colombia; b School of Business Society and Engineering, MDH University, Västerås, Sweden; c Faculty of Management, Department of Engineering Management, AGH University, Kraków, Poland; d Frankfurt Institute for Advanced Studies, Goethe University Frankfurt, Frankfurt am Main, Germany; e Instituto de Pesquisas Hidráulicas (IPH), Universidade Federal do Rio Grande do Sul (UFRGS), Porto Alegre, Rio Grande do Sul, Brazil

**Keywords:** Energetic complementarity, Renewable energy, Variable renewables, Geographic information systems

## Abstract

Renewable energy sources have shown remarkable growth in recent times in terms of their contribution to sustainable societies. However, integrating them into the national power grids is usually hindered because of their weather-dependent nature and variability. The combination of different sources to profit from their beneficial complementarity has often been proposed as a partial solution to overcome these issues. Thus, efficient planning for optimizing the exploitation of these energy resources requires different types of decision support tools. A mathematical index for assessing energetic complementarity between multiple energy sources constitutes an important tool for this purpose, allowing a comparison of complementarity between existing facilities at different planning stages and also allowing a dynamic assessment of complementarity between variable energy sources throughout the operation, assisting in the dispatch of power supplies. This article presents a method for quantifying and spatially representing the total temporal energetic complementarity between three different variable renewable sources, through an index created from correlation coefficients and compromise programming. The method is employed to study the complementarity of wind speed, solar radiation and surface runoff on a monthly scale using continental Colombia as a case study during the year of 2015.

• This paper describes a method for quantifying and spatially representing energetic complementarity between three renewable energy sources.
• The method quantifies energetic complementarity by combining known metrics: correlations and compromise programming.
• The proposed index for energetic complementarity assessment is sensitive to the time scale adopted.

## Graphical abstract

**Figure:**
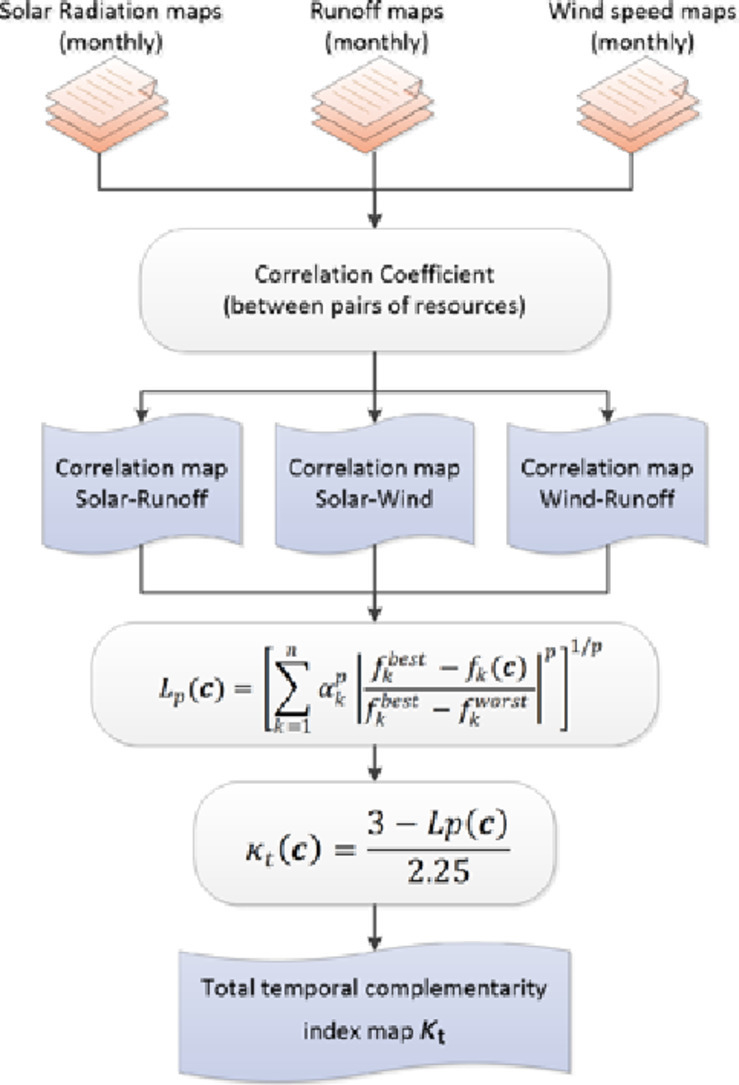

**Specifications Table**

**Table:** 

Subject Area.	Energy
More specific subject area.	*Renewable Energy*
Method name.	*Method for spatially representing temporal complementarity between three variable energy sources based on correlation and compromise programming*
Name and reference of original method.	*Assessing temporal complementarity between three variable energy sources through correlation and compromise programming. Canales et al.**[1]**Energy (2020), v.192, 166637*.
	*Temporal complementarity between three variable renewable energy sources: A spatial representation. Canales et al.**[2]**Proceedings of the 11^th^ International Conference on Applied Energy (2019), Västerås, Sweden.*
Resource availability.	*N/A*

## Method details

### Background

The energy complementarity between renewable resources exploited for the generation of energy in hybrid systems can contribute decisively to the technical and economic viability of these systems, both in stand-alone systems as in interconnected systems. The work of Beluco et al. [3] contributed to a better understanding of energetic complementarity, proposing the differentiation between temporal complementarity and spatial complementarity, proposing the identification of different complementarity components and proposing a dimensionless index [4] to evaluate temporal complementarity. These studies generally dealt with energetic complementarity through an evaluation or comparison of a pair of sources (by means of correlation coefficients or complementarity indices), until the work of Borba and Brito [5], where these authors addressed the problem of assessing the complementarity between more than two energy resources at once,and more recently Han et al. [6] estimated the complementarity between multiple energy sources through the comparison of the fluctuation difference between their individual and combined power generation capacities. Energetic complementarity is a theme that has been concentrating the attention of many researchers around the world in recent years, as is the case of Kougias et al. [7], who proposed a method for orientation of photovoltaic modules aiming at a better global use of the energy available to a PV-hydro hybrid system, and the work of Jurasz et al. [8], which relates the reliability in small hybrid systems to the complementarity between the exploited resources.

Some authors have conducted a spatial representation of energetic complementarity. Silva et al. [9] presented the correlation maps illustrating how hydropower and offshore wind power complement their generation between the different regions of the Brazilian interconnected power system. Vega-Sánchez et al. [10] followed a similar approach in their evaluation of complementarity between wind and solar power resources over Mexico. Cantão et al. [11], prepared hydro-wind correlation maps obtained both from Pearson and Spearman correlation coefficients for the entire Brazil, and they state that this type of information can subsidize operation and expansion strategies for power systems at country scale. The work of Risso et al. [12] presented the concept of “complementary roses” to understand spatial complementarity, and in a follow-up paper, they proposed a qualitative method [13] for assessing spatial complementarity over time based on this model. The previously mentioned paper by Borba and Brito [5] was the only paper found in the literature that presents a spatial representation of a metric assessing complementarity between more than two VRES, extending from the method for estimating a complementarity index, developed by Beluco [3].

This paper presents a method for the spatial representation of an index describing the temporal complementarity between three variable renewable energy sources (VRES). The method is an extension from the paper by Canales et al. [1], for assessing energetic complementarity utilizing a linear metric built on correlation coefficients and compromise programming. For a better understanding, this paper also presents a brief example of the application of the method using reanalysis data for 3 VRES and the continental territory of Colombia for the year 2015 on a monthly scale.

### Method

The method for evaluating the temporal complementarity between three variable energy sources based on correlation and compromise programming consists of the following steps:

1. Determine the data series to be used in the evaluation of temporal complementarity.

Note. Series can be composed of hourly, daily or monthly data. Series shall evenly cover the same period and are named according to the renewable resources to which they are associated, such as `w' for a series of wind speeds, `h' for a series of river flows and `s' for a series of solar energy availability data.

2. Calculate the correlation coefficients between the energy resources taken in pairs and build a vector that will be called ***c***. This vector is the resulting vector of the correlations between each pair of resources taken as unitary vectors in a three-dimensional reference.

Note. Considering three available datasets, one of wind speed, one of streamflow and the other of solar radiation, the correlations between the first and second, between the first and third and between the second and third appear below respectively in Eqs. (1)–(3).
  (1) $$CC_{\text{wh}}=r_{\text{wh}}=\frac{\sum_{i=1}^{n}\left(w_{i}-\bar{w}\right)\left(h_{i}-\bar{h}\right)}{\sqrt{\sum_{i=1}^{n}{\left(w_{i}-\bar{w}\right)}^{2}}\sqrt{\sum_{i=1}^{n}{\left(h_{i}-\bar{h}\right)}^{2}}}=\;=\frac{\text{Covariance}\left(w,h\right)}{\sigma_{w}\sigma_{h}}$$
  (2) $$CC_{\text{ws}}=r_{\text{ws}}=\frac{\sum_{i=1}^{n}\left(w_{i}-\bar{w}\right)\left(s_{i}-\bar{s}\right)}{\sqrt{\sum_{i=1}^{n}{\left(w_{i}-\bar{w}\right)}^{2}}\sqrt{\sum_{i=1}^{n}{\left(s_{i}-\bar{s}\right)}^{2}}}=\;=\frac{\text{Covariance}\left(w,s\right)}{\sigma_{w}\sigma_{s}}$$
  (3) $$CC_{\text{hs}}=r_{\text{hs}}=\frac{\sum_{i=1}^{n}\left(h_{i}-\bar{h}\right)\left(s_{i}-\bar{s}\right)}{\sqrt{\sum_{i=1}^{n}{\left(h_{i}-\bar{h}\right)}^{2}}\sqrt{\sum_{i=1}^{n}{\left(s_{i}-\bar{s}\right)}^{2}}}=\;=\frac{\text{Covariance}\left(h,s\right)}{\sigma_{h}\sigma_{s}}$$

Eq. (4) shows the vector ***c***. Eq. (4) presents a vector ***c,*** which is the vector formed by the components established by the correlations coefficients (CC) between the resources considered in the analysis.

(4) $$c=CC_{\text{wh}}\hat{\text{wh}}+CC_{\text{ws}}\hat{\text{ws}}+CC_{\text{hs}}\hat{\text{hs}}$$

The correlation calculated in Eqs. (1) to (3) computes the Pearson correlation coefficient. There are other methods for determining the correlation, as discussed by Canales et al. [3].

3. Determine the parameter Lp as a function of vector ***c***, as defined in Eq. (5).
  (5) $$L_{p}\left(c\right)={\left[\sum_{k=1}^{n}\alpha_{k}^{p}{\left|\frac{f_{k}^{\text{best}}-f_{k}\left(c\right)}{f_{k}^{\text{best}}-f_{k}^{\text{worst}}}\right|}^{p}\right]}^{1/p}$$
  Note. In Eq. (5) $\alpha_{k}^{p}$ are the relative weights for each component k (where k is each paired combination). If all paired combinations are considered as equally important, all $\alpha_{k}^{p}\;=$ 1. The f_k_(**c**) represents the CC value for the corresponding paired combination of sources in vector ***c***; $f_{k}^{\text{best}}$ is the most desirable value of the correlation functions, thus $f_{k}^{\text{best}}=-\;1$, represent full complementarity as explained in Cantão et al. [11]; $f_{k}^{\text{worst}}$ is the least desirable value of the correlation functions, thus $f_{k}^{\text{worst}}=1$, representing full similarity [11]; p is the parameter determining the type of geometrical distance between $f_{k}^{\text{best}}$ and f_k_(**c**). Gershon and Duckstein [14] explained that *p* = 1 indicates that all deviations from $f_{k}^{\text{best}}$ are considered in direct proportion to their magnitudes, and that *p* = 2 represents the Euclidean distance. The authors suggest adopting the value of *p* = 1, for a linear assessment of temporal complementarity.
4. Determine the temporal complementarity index κ_t_ as a function of Lp, through Eq. (6):
  (6) $$\kappa_{t}\left(c\right)=\frac{3-Lp\left(c\right)}{2.25}$$
  Note. The normalization through Eq. (6) allows a linear scale of these κt values, ranging from 0 (representing perfect similarity) to 1 (representing perfect complementarity), whose interpretation can be expressed in terms of the normalization of the correlation coefficient, as shown in Table 1.

**Table 1:** Interpretation of correlation coefficient values.

Behavior	*κ_t_*(*c*)	Interpretation
Similarity	0.00 ≤ Norm. (CC) < 0.05	Very strong similarity
	0.05 ≤ Norm. (CC) < 0.20	Strong similarity
	0.20 ≤ Norm. (CC) < 0.35	Moderate similarity
	0.35 ≤ Norm. (CC) < 0.50	Weak similarity
Complementarity	0.50 ≤ Norm. (CC) < 0.65	Weak complementarity
	0.65 ≤ Norm. (CC) < 0.80	Moderate complementarity
	0.80 ≤ Norm. (CC) < 0.95	Strong complementarity
	0.95 ≤ Norm. (CC) < 1.00	Very strong complementarity

The minimum achievable Lp(c) value in Eq. (5) is 0.75, as demonstrated in the demonstration show in the development of Eq. (7):

The existence of n random variables x_i_, each with variance = 1 (as in the case of Pearson's correlation coefficient) results in:

(7) $$\begin{array}{ccc} Var\left(\sum_{i=1}^{n}x_{i}\right) & = & \sum_{i,j=1}^{n}Cov\left(x_{i},x_{j}\right)\\ Var\left(\sum_{i=1}^{n}x_{i}\right) & = & \sum_{i=1}^{n}Var\left(x_{i}\right)+\sum_{i\neq j}Cov\left(x_{i},x_{j}\right)\\ Var\left(\sum_{i=1}^{n}x_{i}\right) & = & n+\sum_{i\neq j}\rho_{i,j}\\ Var\left(\sum_{i=1}^{n}x_{i}\right) & = & n+\frac{2n!}{2\left(n-2\right)!}\bar{\rho }\\ Var\left(\sum_{i=1}^{n}x_{i}\right) & = & n+n\left(n-1\right)\bar{\rho } \end{array}$$

Besides, the condition $Var(\sum_{i=1}^{n}x_{i})$ ≥ 0, leads to:

(8) $$\bar{\rho }\geq -\frac{1}{n-1}$$

In consequence, for *n* = 3, it results in $\bar{\rho }\geq -0.5$, therefore Lp(c) ≥ 0.75.

### A brief example – spatial representation of complementarity between surface runoff, solar irradiation and wind speed in Colombia for the year 2015

**Fig. 1:**
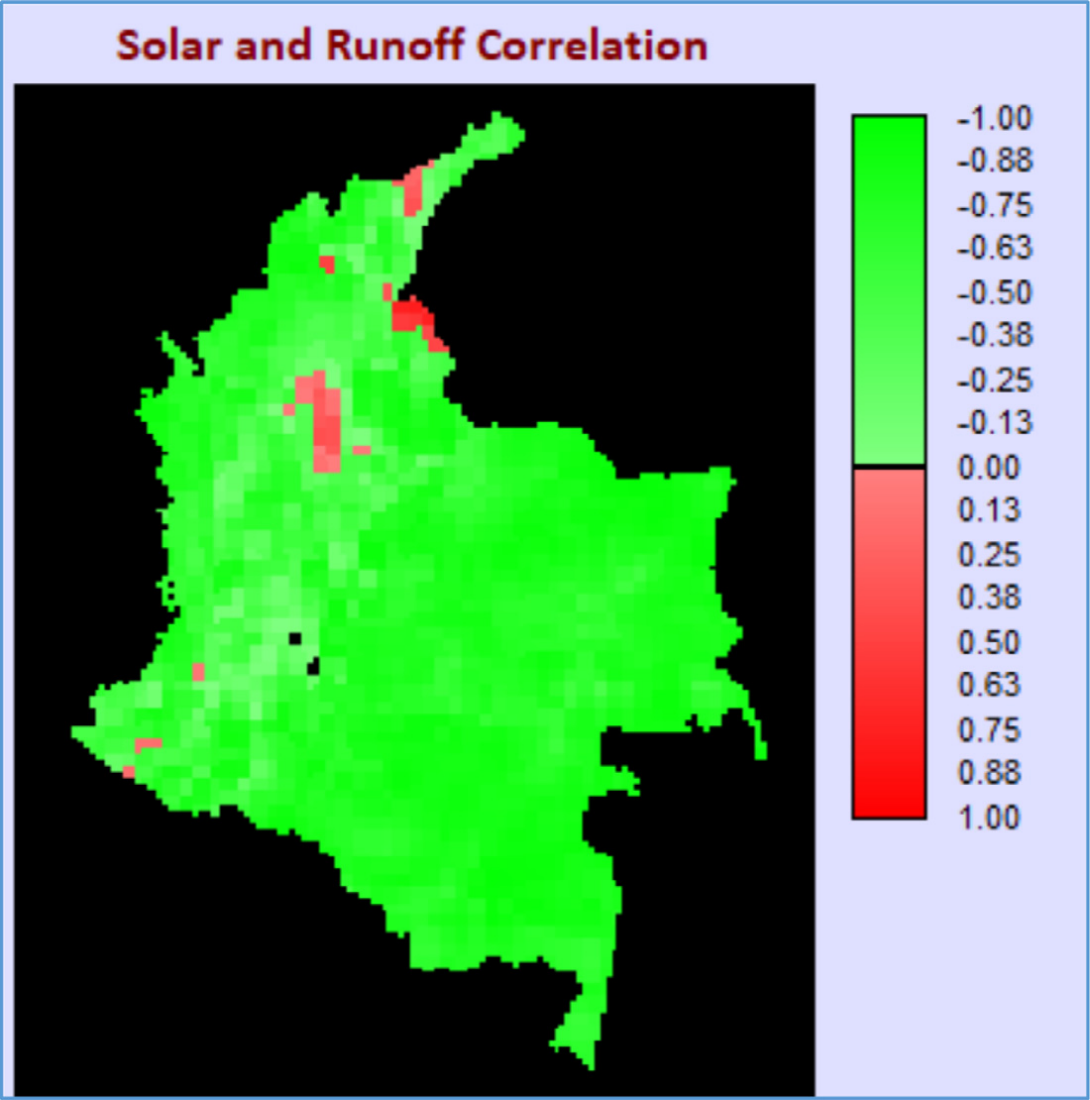
Correlation map for radiation and surface runoff.

**Fig. 2:**
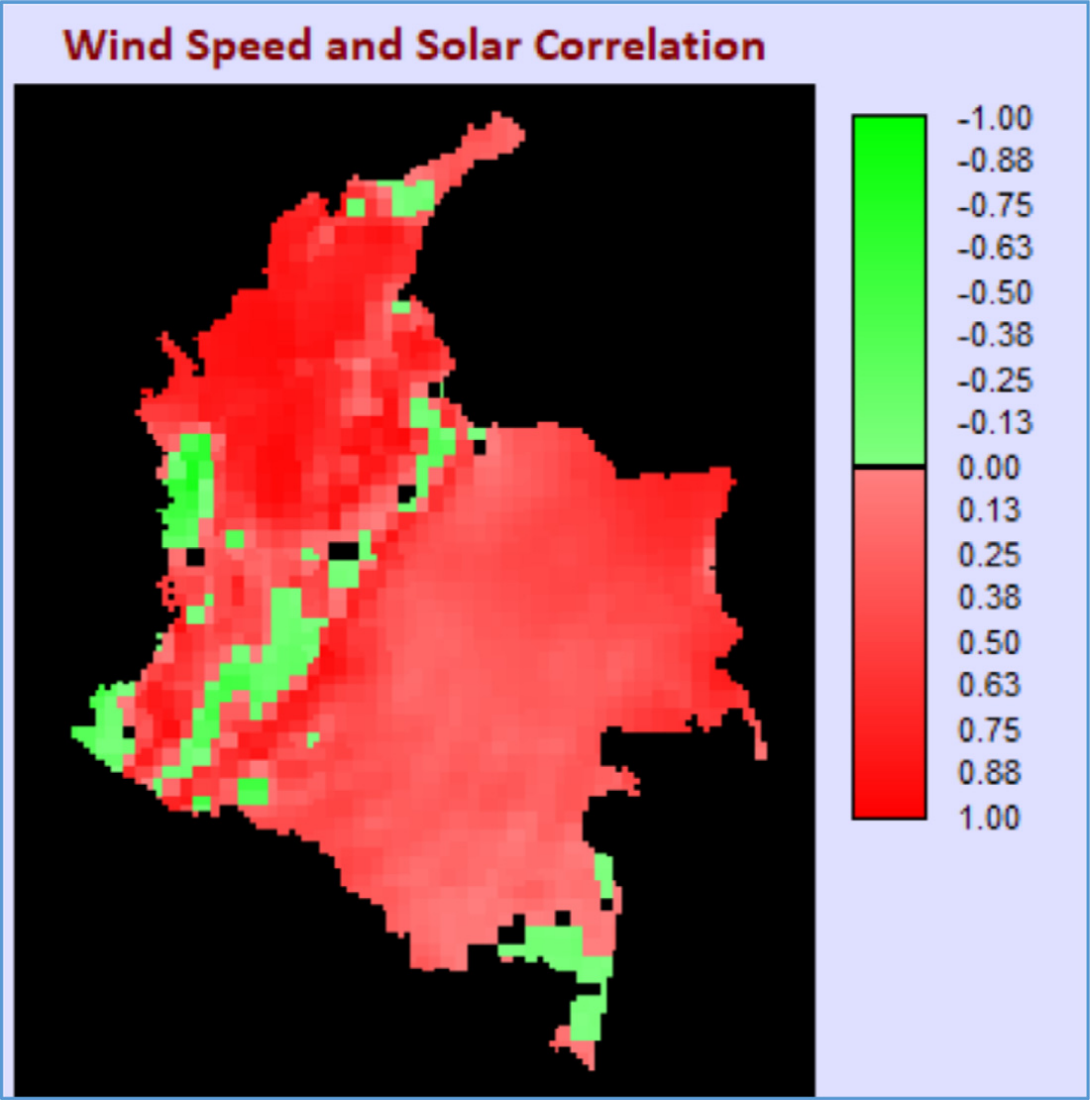
Correlation map for radiation and wind speed.

**Fig. 3:**
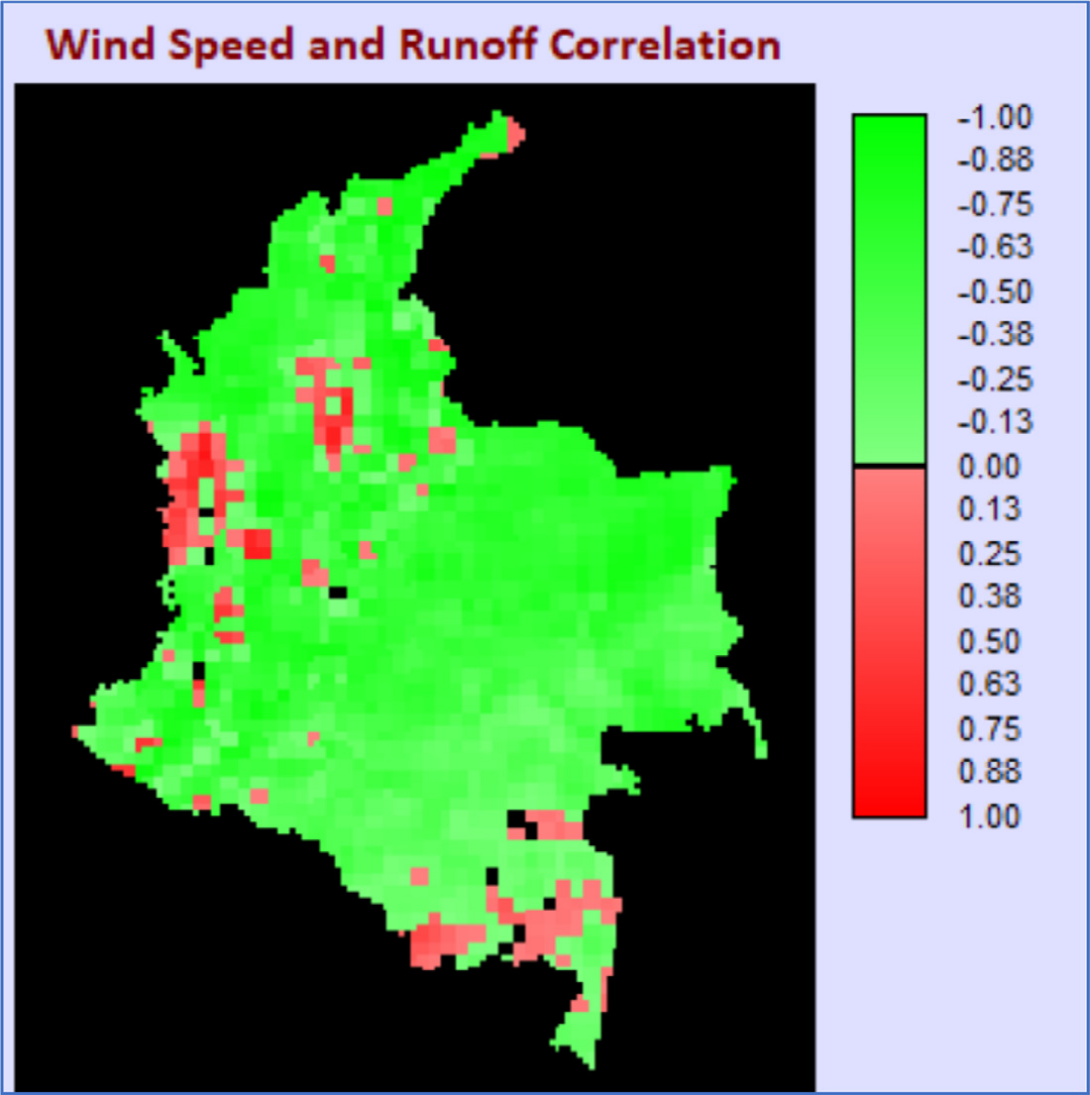
Correlation map for surface runoff and wind speed.

Situated in the northern part of South America, the continental territory of Colombia was used as a case study (approx. 1,142,748 km²) for this paper. The dataset used in this work corresponds to the average monthly solar net radiation, surface runoff and wind speed at 10 m from ERA5 reanalysis [15] for the year of 2015. As a result of employing the data and methods previously described, the correlation for each paired combination of resources is displayed in Fig. 1, Fig. 2, Fig. 3.

The correlation map in Fig. 1 indicates most of the country presents a complementary behavior between solar radiation and surface runoff. However, it is worth mentioning that this does not automatically mean that hydropower potential is available in all regions, because this generation capability also depends on the available head and other favorable and specific terrain features.

The wind speed and solar radiation correlation map (Fig. 2) shows that most of the territory exhibits a similar behavior between these two VRES, except for some regions in the West and South of the country.

Similar to solar radiation and surface runoff, most of the country presents a complementary behavior along the year for wind speed and surface runoff (Fig. 3).

**Fig. 4:**
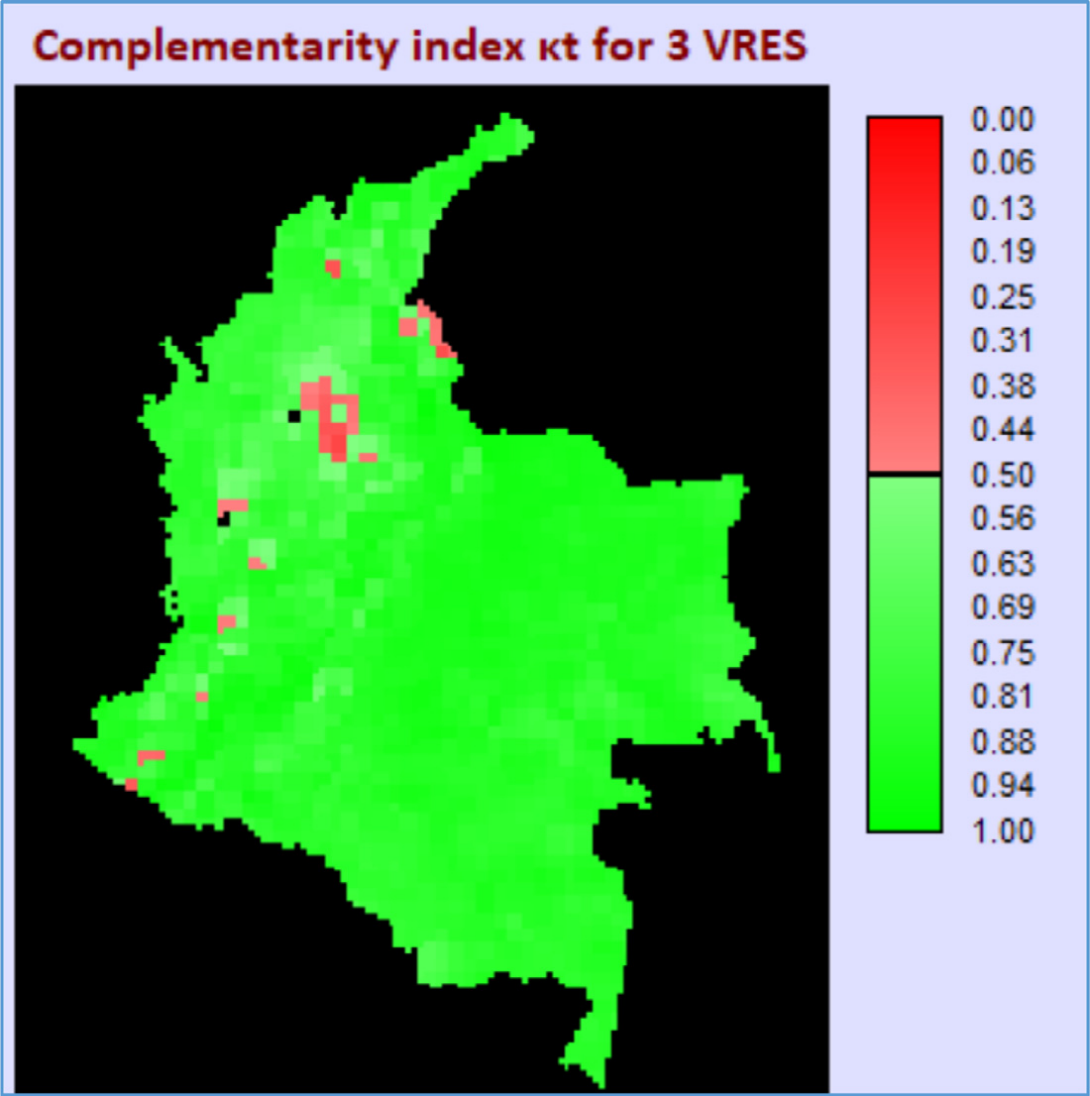
Map showing KT index at each location.

Once the three correlation maps are found, Eqs. (5) and (6) are applied, resulting in the map shown in Fig. 4. This map presents the κt values for the entire territory under consideration.

**Fig. 5:**
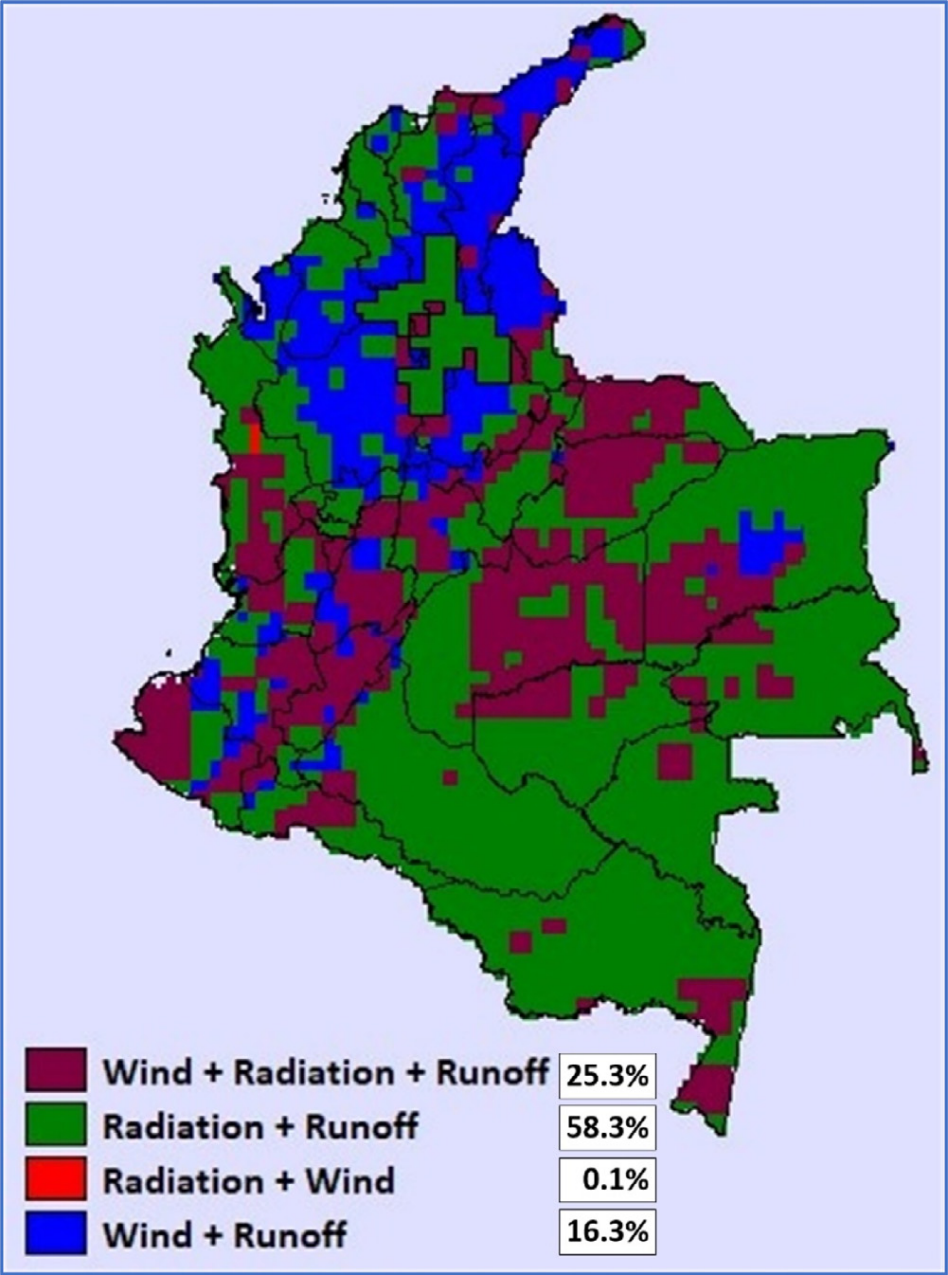
Map of best options in terms of energetic complementarity on a monthly scale.

Normalizing the correlation values within a scale from 0 (full similarity) to 1 (full complementarity) allows estimating which one of the four possible combinations presented from Figs. 1 to 4 is the best option at each location. The map in Fig. 5 shows the results from this normalization, with results suggesting that, in terms of complementarity, the higher normalized score for most of the country comes from the correlation between radiation and runoff, followed by the complementarity between the 3 VRES.
